# Photocatalytic Edge Growth of Conductive Gold Lines On Microstructured TiO_2_–ITO Substrates

**DOI:** 10.1021/acs.langmuir.4c02106

**Published:** 2024-08-28

**Authors:** Fatemeh Abshari, Salih Veziroglu, Blessing Adejube, Alexander Vahl, Martina Gerken

**Affiliations:** † Chair for Integrated Systems and Photonics, Department of Electrical and Information Engineering, Faculty of Engineering, Kiel University, Kaiserstr. 2, D-24143 Kiel, Germany; ‡ Chair for Multicomponent Materials, Department of Materials Science, Faculty of Engineering, Kiel University, Kaiserstr. 2, D-24143 Kiel, Germany; § Kiel Nano, Surface and Interface Science KiNSIS, Kiel University, Christian-Albrechts-Platz 4, D-24118 Kiel, Germany; ∥ Leibniz Institute for Plasma Science and Technology, Felix-Hausdorff-Str. 2, 17489 Greifswald, Germany

## Abstract

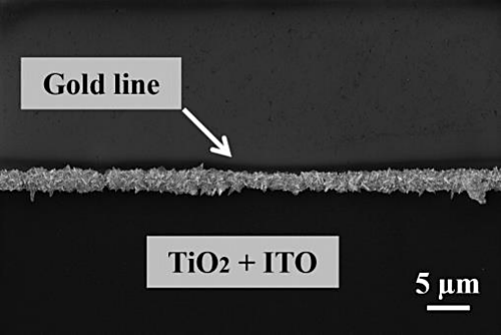

Titanium dioxide is well-known for its excellent photocatalytic properties. UV-controlled photodeposition of gold on TiO_2_ is achieved by photocatalytic reduction of precursor ions from a tetrachloroauric solution. During the growth process on the surface, clusters grow from nucleation centers and coalescence is observed for sufficiently long UV illumination times, resulting in gold structures with complex shapes. Here, we hypothesize and demonstrate that the growth process is altered by employing an ITO sublayer below the TiO_2_ layer. Photocatalytic gold growth experiments on a microstructured thin film stack of 6 nm ITO and 70 nm TiO_2_ lead to strongly localized gold growth along the edge of the patterned area. A conductive gold line with a height of 3.8 μm is achieved along the edge of the TiO_2_-coated region, while gold growth on the surface of TiO_2_ is effectively suppressed. For substrates coated only with ITO or TiO_2_, no edge growth is observed. Furthermore, for an 845 nm thick TiO_2_ layer, either with or without ITO sublayer, gold growth on the TiO_2_ surface is dominant. Thus, for the effective steering of electrons to the edge, both the ITO sublayer and a sufficiently thin TiO_2_ layer are necessary. This modified method of photocatalytic deposition—electrons photogeneration in a thin layer, collection in a dedicated conductive sublayer, and growth by reduction at a different position—opens opportunities for localized material deposition. We are in particular aiming at extending the toolbox of neuromorphic engineering by providing a technical implementation of stimulus-controlled dynamic formation of directional conductive interlinks.

## Introduction

Neuromorphic engineering aims to develop efficient computing approaches inspired by biological neural networks.^1^ Synaptic connections between individual neurons in a neural network are reconfigured dynamically. These connections develop over different time scales: Fast synaptic plasticity involves changes at the local level of synaptic connections between two neurons, whereas slow blooming and pruning take place globally throughout the neural network. Many studies have focused on mimicking the fast synaptic plasticity using memristive devices owing to their unique capability of in-memory computing.^2^ Investigation of synaptic connections at a global scale in biological neural networks as well as development of efficient approaches to integrate them into future bioinspired systems remain ongoing areas of research.^3^ Recently, neuromorphic nanowire networks with memristive properties have been studied.^4−6^ These networks are self-organized, with the nanowires as one-dimensional (1D) conductive pathways. Collective switching properties arise from a complex network topology suitable for memristive architectures. In neural networks, the capacity to dynamically regulate stimuli and control the formation and dissolution of network connections is a crucial factor.^7^ Mimicking the global interactions of neuron assemblies was initially achieved by investigating global connectivity through electrolyte gating within a liquid medium.^8^ In this paper, our focus lies on the slow-growing formation of 1D long-range connections that are potentially suitable for the on-demand adaptability of a network topology. For this purpose, we investigate the photocatalytic deposition of conductive gold lines from solution on UV-stimulated TiO_2_.

TiO_2_ has been widely utilized as a semiconductor photocatalyst owing to its remarkable properties including excellent photocatalytic properties, simple and cost-effective processing, nontoxicity, and chemical inertness.^9−12^ Gold growth on the surface of TiO_2_ thin films has been demonstrated by photoreduction of HAuCl_4_ in the presence of UV light.^13−15^ Numerous studies have focused on investigating the factors that influence the morphology of the resulting gold structures. In addition to the crystal structure and morphology of the underlying TiO_2_ thin film, the composition and pH of the precursor solution, illumination intensity, and duration play an important role in determining the morphology and coverage of the deposited gold structures.^16−18^ Our recent study^18^ revealed that UV illumination time and intensity significantly influence the growth and morphology of Au clusters on TiO_2_ thin films. The growth process begins with the formation of stable Au nuclei on the TiO_2_ surface. Extended UV exposure facilitates the reduction of additional Au^3+^ ions, resulting in a larger cluster formation. Higher UV intensity accelerates Au cluster nucleation and growth, leading to needle-like structures. This results in locally increased electric fields and electron densities at the sharp tips, promoting the preferential reduction of Au^3+^ ions in these regions. Here, we demonstrate that the UV illumination time affects the width and height of the gold lines. Longer UV illumination times lead to more photocatalytic reduction of HAuCl_4_ on the TiO_2_–ITO substrate. Over time, we observe the growth of larger clusters and coalescence to a conductive gold line.

The photocatalytic deposition of gold nanoparticles on a TiO_2_ thin film with a columnar morphology was demonstrated under ultraviolet (UV) light irradiation using a gold precursor solution.^19^ Lateral selectivity in the deposition of metallic structures has been achieved by selective illumination using shadow masks and by patterning the underlying TiO_2_ thin film using lithography techniques.^7,16,18^ In a recent study, Au/TiO_2_–C_3_N4 composites with plasmonic gold nanoparticles were developed, showing superior photocatalytic performance due to the high visible light absorption and prolonged lifetime of photoexcited charge carriers.^20^ A similar work has demonstrated the coalescence of gold clusters under UV light, emphasizing photon energy-dependent pathways that influence photocatalytic behavior. These findings provide additional context for understanding the role of UV illumination in the growth and morphological modification of gold particles.^21^

Here, we utilize photocatalysis to grow gold lines on TiO_2_ thin films patterned by lithography. The addition of a thin layer of indium tin oxide (ITO) below TiO_2_ is investigated. Owing to the higher work function of ITO compared to TiO_2_, a Schottky barrier is formed at the TiO_2_–ITO interface.^22,23^ We hypothesize that the transfer of photogenerated electrons from TiO_2_ to ITO changes the photocatalytic growth process on the microstructured TiO_2_–ITO substrates. In this study, the gold growth is investigated experimentally with and without the ITO sublayer and two TiO_2_ layer thicknesses.

## Experimental Section

### Substrate Preparation

**Figure 1:**
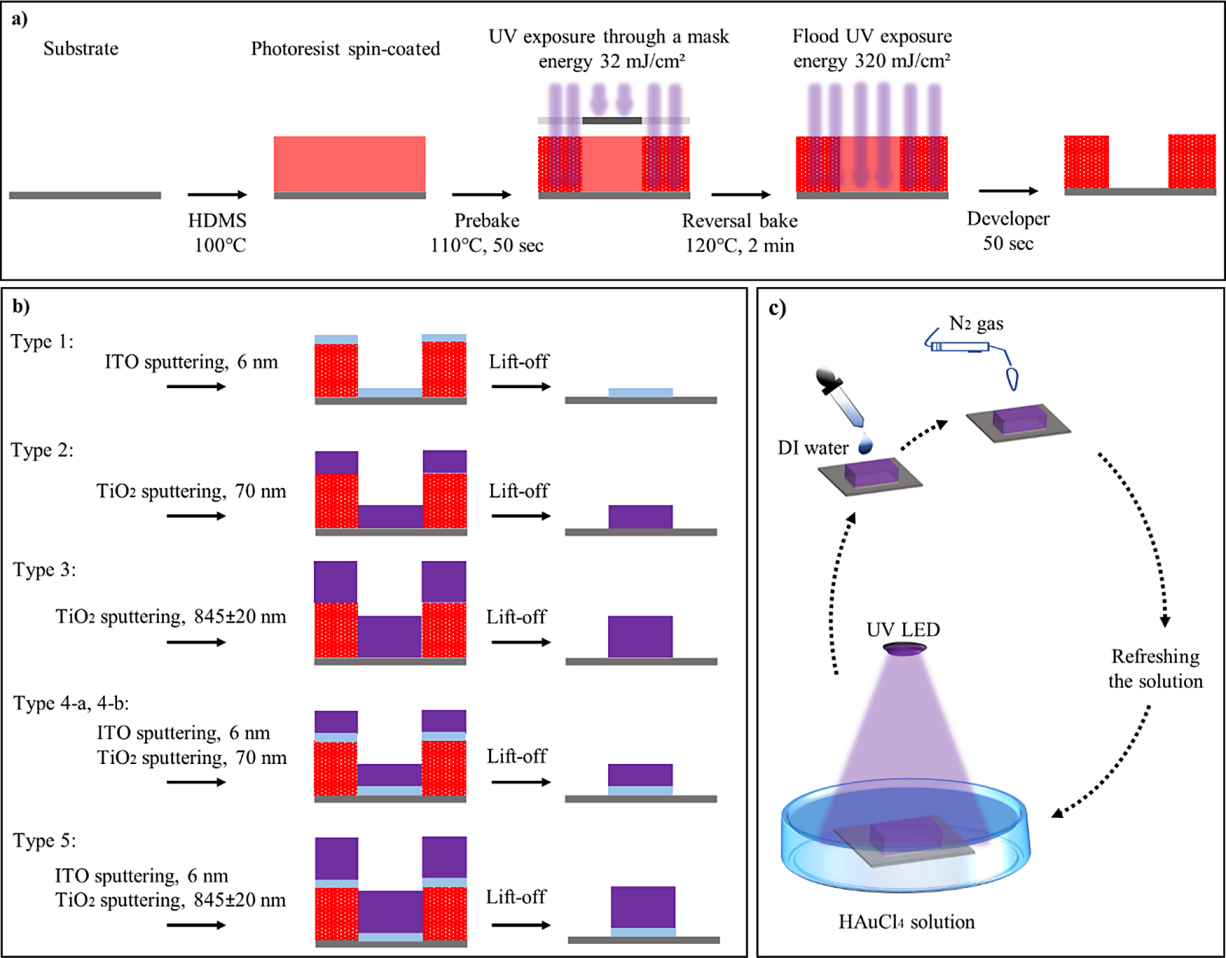
Schematic illustration of the substrate preparation process and the growth illumination setup. (a) Lithography process steps using AZ5214E photoresist on a Si wafer substrate with a 1 μm thermal oxide layer. (b) Illustration of the sputtering and lift-off steps, presenting resulting substrate patterns (type 1: ITO (6 nm), type 2: TiO_2_ (70 nm), type 3: TiO_2_ (845 nm), types 4-a and 4-b: ITO (6 nm) and TiO_2_ (70 nm) and type 5: ITO (6 nm) and TiO_2_ (845 nm). (c) Schematic representation of the cyclic photocatalytic growth experiment. Each cycle begins with UV illumination, followed by substrate washing with deionized water, drying with N_2_ gas, and refreshing the solution, leading to another round of illumination.

UV photolithography was used to create microstructures on silicon wafers, as illustrated in Figure 1a. Five types of substrates, designated as types 1–5, were subsequently prepared by physical vapor deposition (PVD) of ITO and/or TiO_2_ coatings. To obtain the type 1 substrate, a thin layer of ITO (6 nm) was deposited, whereas the type 2 substrate was created by deposition of a 70 nm thick TiO_2_ layer. The type 4-a and type 4-b substrates were fabricated by depositing a 6 nm layer of ITO followed by a 70 nm layer of TiO_2_. Then a lift-off process was carried out to remove the remaining photoresist together with the ITO or TiO_2_ layers on top of it. The fabrication processes of different substrate types, including sputtering and lift-off steps, are summarized in Figure 1b. To convert the TiO_2_ thin films to the anatase phase, the substrates underwent a heat treatment process. To achieve thicker TiO_2_ with a different morphology, a second TiO_2_ sputtering method was exploited for the type 3 and type 5 substrates (see Supporting Information for details).

### Photocatalytic Gold Growth Experiment

In this section, the photocatalytic gold growth experiment is detailed. The photocatalytic reduction of precursor ions from a HAuCl_4_ solution was achieved by utilizing UV-illuminated TiO_2_ and ITO patterns. To prepare the precursor solution, 99.99% pure Gold(III) chloride powder (Sigma-Aldrich) was carefully mixed with deionized water in a ratio of 15 mg to 60 mL. The components were thoroughly blended to achieve a homogeneous solution. The substrate was positioned at the bottom of a glass beaker with a diameter of 5 cm, and 15 mL of the prepared solution was added subsequently. Above the beaker, a UV LED (Nichia) with a wavelength of 365 nm was positioned at a distance of ∼7 cm to provide an intensity of ∼3.7 mJ/cm^2^ (measured using a Newport optical power meter). The illumination process on the type 1, 2, 3, 4-a, and 5 substrates was conducted in two steps: an initial 150 min of illumination, followed by refreshing the precursor solution and another 60 min of illumination. The type 4-b substrate underwent a three-step illumination, with each round lasting 150 min. After each illumination round, the substrate was thoroughly washed and dried. Figure 1c schematically illustrates the cyclic illumination of the substrates in the photocatalytic growth experiment, and Table 1 summarizes the key parameters for each of the samples.

**Table 1:** Material Compositions and Illumination Times for Each Type of Substrate^a^

		illumination time	
		150 min, 60 min	3 × 150 min
Material composition	ITO	Type 1	
	TiO_2_	Type 2 and 3	
	ITO/TiO_2_	Type 4-a and 5	Type 4-b

a Type 4-b substrates were illuminated three times for 150 min. All other substrates were first illuminated for 150 min followed by a second 60 min illumination.

### Sample Characterization after Gold Growth

After the gold growth, the morphology and chemical composition of the samples were examined using scanning electron microscopy (SEM, Supra55VP-Carl Zeiss) at an acceleration voltage of 3 kV (with a 3 mm working distance) and EDX (Oxford Instruments, Ultim Max 65). Additionally, atomic force microscopy analysis in tapping mode (cantilever description: spring constant 2 N/m and resonance frequency ∼70 kHz) was carried out utilizing an atomic force microscope (Renishaw, MODEL alpha300 A) equipped with a Leica DM2500 microscope. Conductance measurements were performed using the Everbeing BD-6 modular probe station, equipped with micromanipulators for precise contacting, a PSM-1000 microscope, and a Motic Moticam 3+ CCD camera for high-quality image acquisition. A Keithley 2400 Sourcemeter, configured in a two-contact setup, facilitated accurate and reliable conductance measurements by recording the current response to voltage ramps.

## Results and Discussion

### Morphological Characterization via SEM

**Figure 2:**
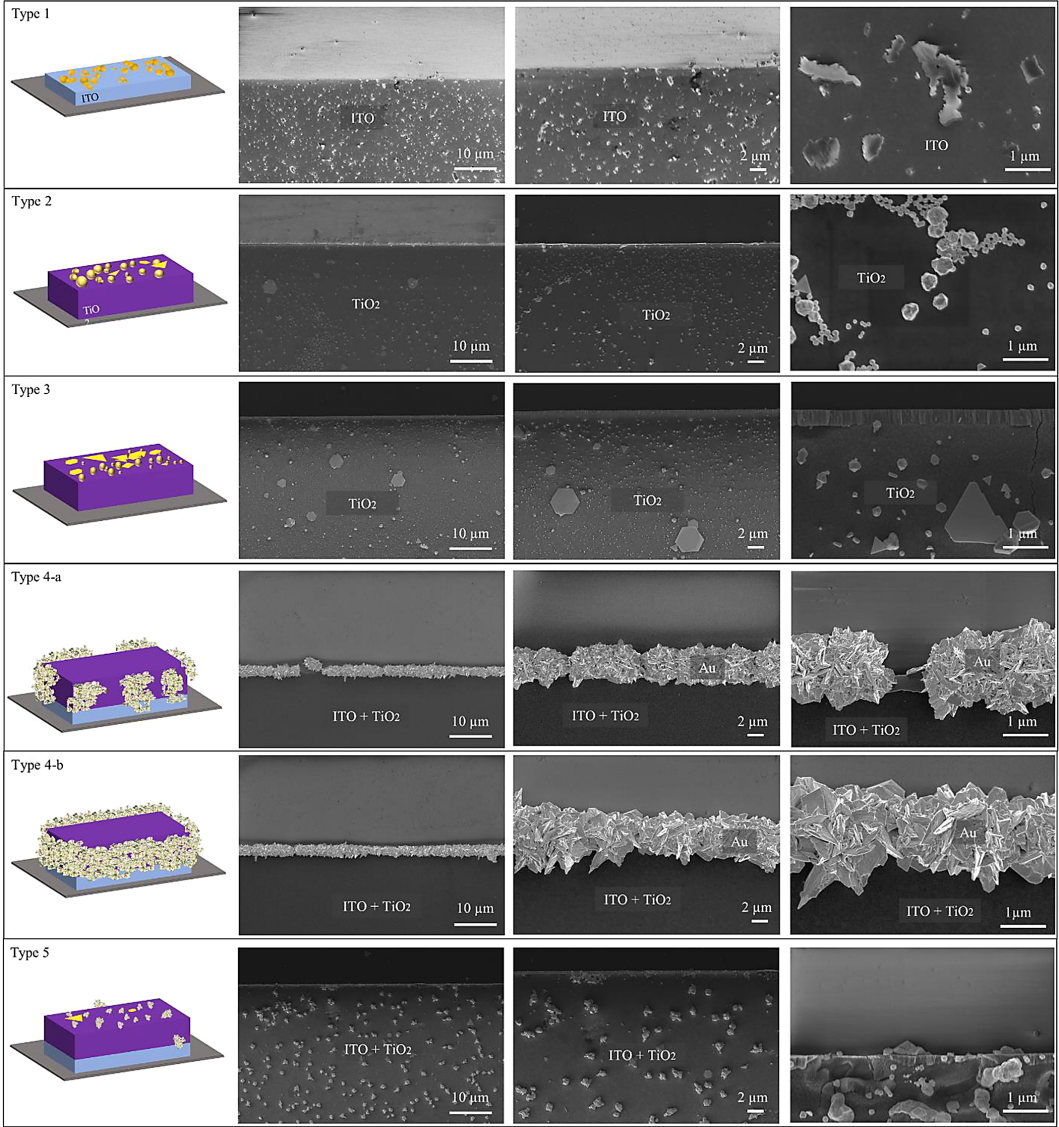
SEM images of the five different types of substrates (type 1–type 5), as listed in Table 1, following gold growth. In each of the SEM images, an edge between the uncoated wafer, i.e., the upper section of each image, and the coated wafer, i.e., the lower section, is visible. The 3D schemes on the left side visualize the structural details of each substrate.

For each substrate type 1–5, several samples were fabricated and analyzed. Figure 2 shows representative SEM images of the different types of substrates after the photocatalytic gold growth process. SEM images of two different samples of each substrate type are included in Figure S1. In the first row of Figure 2, SEM images of type 1 substrates are shown. The SEM images are taken such that an edge between the uncoated wafer, i.e., the brighter region in the upper section of the image, and the ITO-coated wafer, i.e., the darker region in the lower section, is visible. The formation of randomly distributed gold islands with dimensions less than 1 μm is observed on the ITO surface.

The type 2 substrates exhibit island growth of gold on the TiO_2_-coated regions, and some spherically grown structures are also observed. Larger spherical structures, with an average dimension of approximately 400 nm, and smaller spheres, averaging around 120 nm in size, are observed. Furthermore, some of the grown particles take on shapes that resemble triangles and polygons. One can observe instances of superposition in certain areas, wherein spherical particles are found on top of planar particles. The edge appears brighter in the SEM image, but does not show enhanced gold growth.

The type 3 substrates also show gold growth in the TiO_2_-coated region. The morphology of the grown particles reflects that of the type 2 substrate, featuring two main categories: spherical and polyhedral planar particles. Notably, the three-dimensional structure of spherical particles is more prominently visible on these substrates. The grown polyhedral planar particles exhibit triangular and polygonal forms, with instances of superposed stacks evident in SEM images. For none of the type 1 to 3 substrates, the photodeposited gold structures reach the percolation threshold within the illumination time of 210 min.

A very different growth situation is observed for the type 4-a and 4-b substrates with a thin TiO_2_–ITO layer and two different UV illumination times. On these substrates, gold growth is solely observed at the edge between the uncoated and the coated region. No considerable gold growth is observed on the surface of the TiO_2_–ITO thin film stack apart from the edge region. The morphology of the grown particles on the type 4 substrates differs significantly from the previous observations. Instead of spherical and planar formations, flower-shaped structures are formed at the edges. These structures consist of crossed plates of grown gold closely arranged to create a uniform line. For the type 4-b substrate with a total illumination time of 450 min, a continuous gold line is formed along the edge of the microstructured TiO_2_–ITO thin film stack.

SEM images of type 5 substrates with a thick TiO_2_ layer on ITO reveal that particles are mostly grown on the surface. Three of the four investigated type 5 substrates show only little growth along the edge, whereas more gold growth along the edge is observed for one substrate (see Figure S2). The morphology of the particles on the TiO_2_ surface resembles that of type 2 substrates. While planar particles are also observed, the distinguishing feature lies in the spherically grown particles. Nanostars are formed here, featuring a 3D structure with needle-like protrusions.

**Figure 3:**
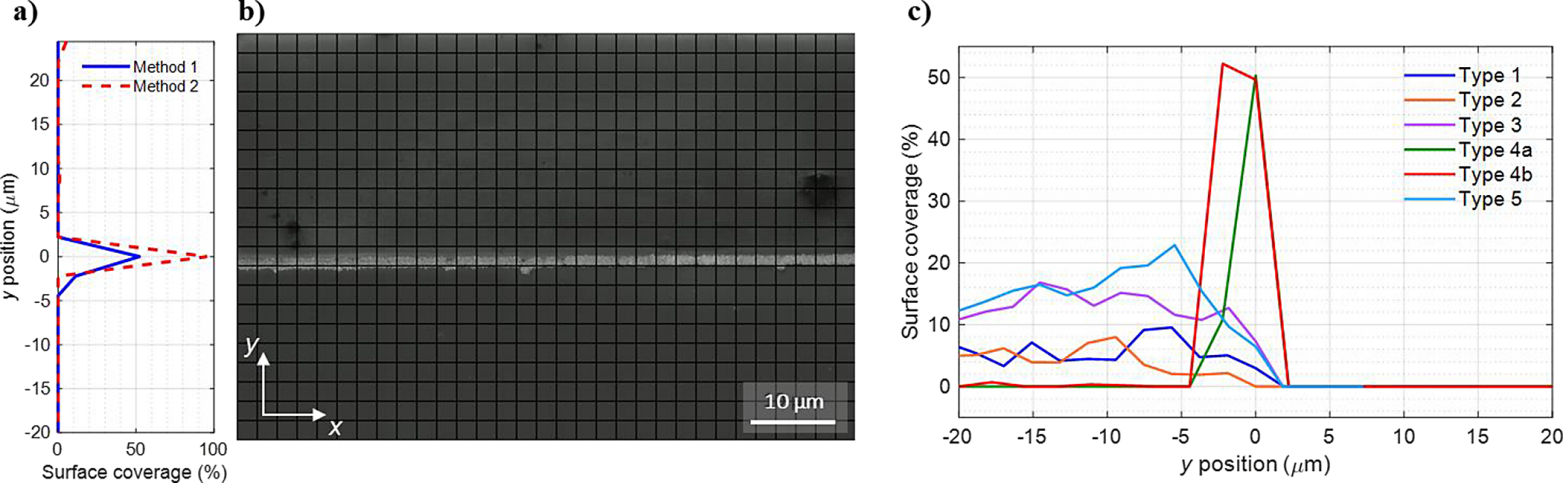
Evaluation of the gold surface coverage (see Figure S2 for details). (a) Averaged surface coverage per row for the SEM image in (b) is plotted. Method 1 uses manual estimation of surface coverage with an overlaid grid on the SEM image as seen in (b). Method 2 exploits an automated image evaluation. (c) Surface coverage results obtained by manual counting for all of the different substrate types. The edge is positioned at *y* = 0.

From the SEM images, the gold surface coverage for the different substrate types was estimated. For this purpose, two different methods were implemented: (1) manual counting and (2) automated image analysis. Figure 3b showcases the SEM image of a type 4 substrate overlaid with a grid for manual counting. The gold surface coverage was estimated within each section of the displayed grid, and the acquired values were integrated in the *x*-direction. This results in the surface coverage plot as a function of the *y*-position depicted in Figure 3a. The edge between the uncoated and coated wafers is set to *y* = 0. The procedure is repeated for all substrate types, and more data are given in the Supporting Information.

As previously observed, the type 4 substrates exhibit a distinct behavior compared to the other substrates with dominant gold growth along the edge and insignificant growth on the TiO_2_-coated surfaces. For the other substrate types, however, significant gold growth is observed on the TiO_2_-coated surfaces. The gold coverage on the surface away from the edge, i.e., from *y* = −20 to *y* = −10 μm in Figure 3c, is summarized in Table 2. It is highest for the type 3 and type 5 substrates with approximately 12 and 15% gold coverage on TiO_2_ surface, respectively. The type 1 and type 2 substrates exhibit roughly 5% surface coverage, whereas the coverage away from the edge in the type 4-a and 4-b substrates is almost negligible.

**Table 2:** Surface Coverage (%) of Gold on the TiO_2_-Coated Substrate Away From the Edge^a^

substrates	type 1	type 2	type 3	type 4-a	type 4-b	type 5
surface coverage (%)	5.2	5.7	12	0	0.2	15

a The coverage is averaged for the section from *y* = −20 to *y* = −10 μm in Figure 3c.

### Morphological Characterization via AFM Analysis

**Figure 4:**
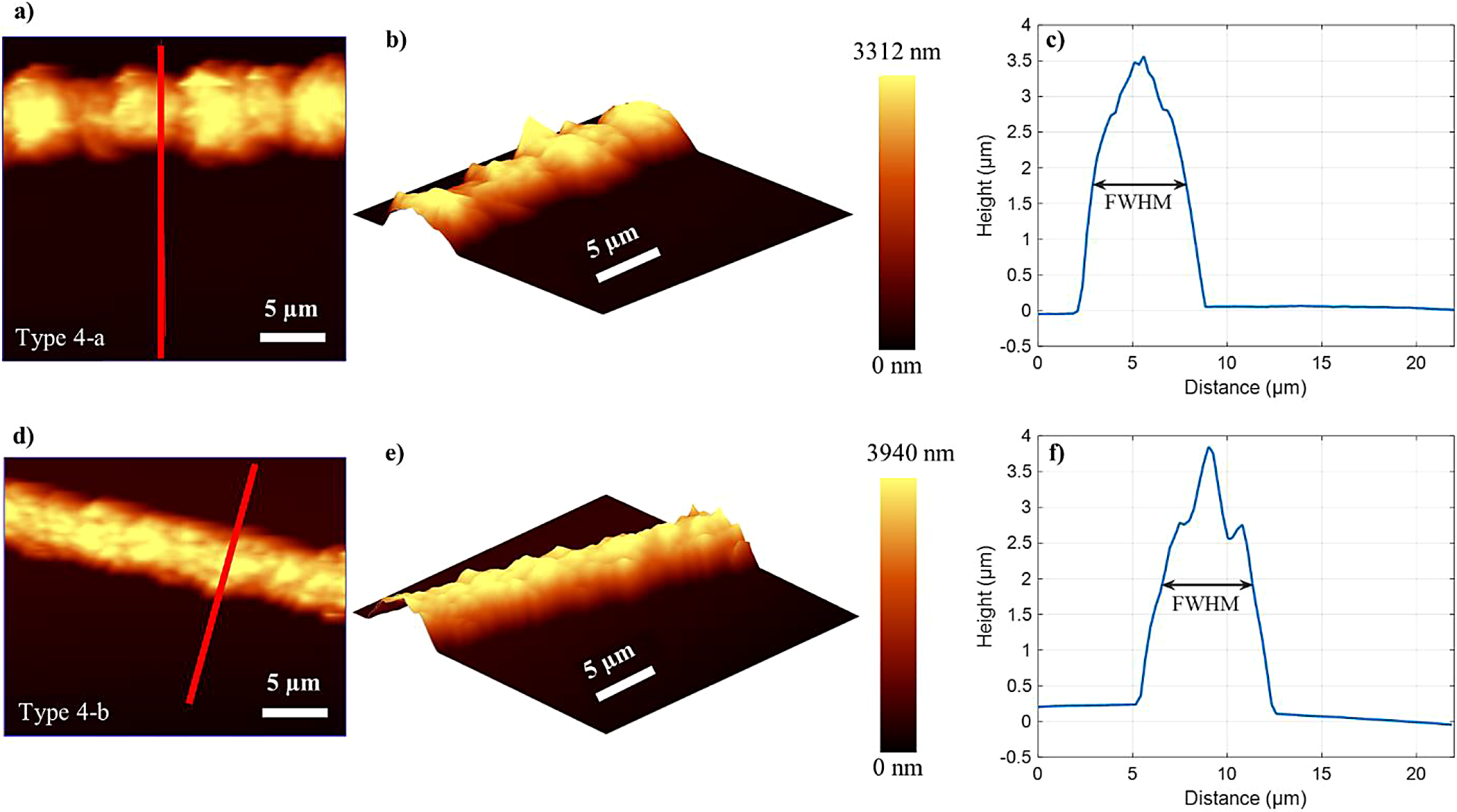
AFM images of the grown gold lines on the (a, b) type 4-a and (d, e) type 4-b substrates. (a, d) Top view topography images, (b, e) 3D visualizations, and (c, f) height profiles extracted along the red lines indicated in (a) and (d).

In this section, we investigate the topography images obtained from atomic force microscopy and perform 3D visualization of the grown gold lines on type 4-a and 4-b substrates. Both substrates share a common structure, featuring the combination of ITO and TiO_2_ patterns. The distinguishing factor lies in the variation of the illumination time during the photocatalytic growth process. The AFM analysis generates detailed topography images showcasing the surface features of the gold lines grown on the type 4-a (Figure 4a,b) and type 4-b (Figure 4d,e) substrates. Additionally, height profiles are extracted along the red lines indicated in Figure 4a,d, depicting the approximate height of the grown lines on each of the two substrates. For type 4-a, a peak height of ∼3.5 μm and a full width at half-maximum (fwhm) of ∼4.9 μm are obtained for the grown gold line as depicted in Figure 4c. For the type 4-b substrate with a longer illumination time, a height of ∼3.8 μm and a fwhm of ∼4.7 μm are observed. The type 4-a substrate shows a higher spatial variability of the height along the grown line compared to the type 4-b substrate.

### Characterization of Chemical Composition via EDX Analysis

**Figure 5:**
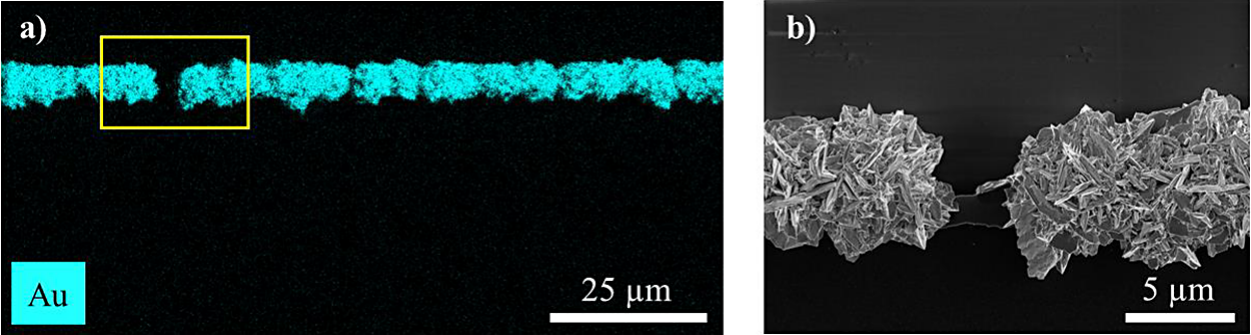
(a) EDX elemental map of a type 4-a substrate, showing the distribution of Au. (b) SEM image of the marked area in (a) with a gap in the grown Au line.

Energy-dispersive X-ray spectroscopy (EDX) was carried out to investigate the elemental composition of the lines grown on type 4 substrates. In Figure 5a the EDX elemental map highlights the distribution of gold (Au), providing clear evidence that the grown line is indeed composed of gold. Furthermore, the absence of gold signals in the areas away from the edge is noted, confirming that growth is concentrated at the edge for these samples. For a better understanding of the line’s morphology, Figure 5b presents the SEM image of the marked region in Figure 5a, revealing a gap within the grown gold line. Additional EDX results are provided in Figure S3 for more detailed analysis.

### Conductance Measurement

**Figure 6:**
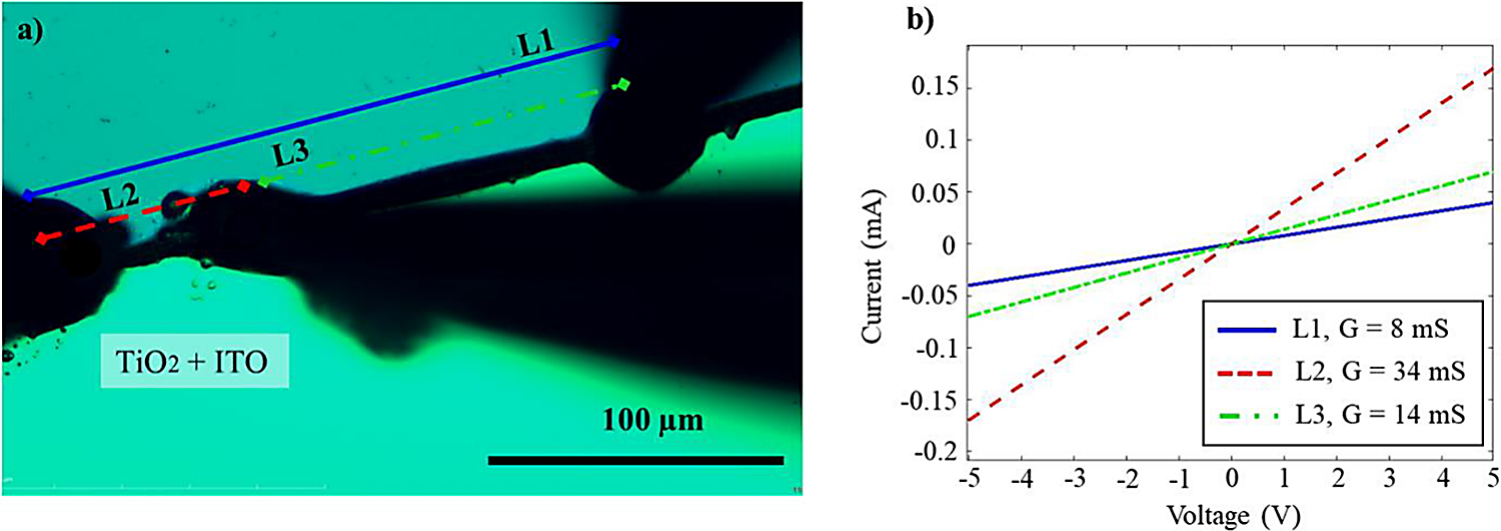
Conductance measurement of gold lines. (a) Optical microscopy image of the type 4-b substrate featuring gapless grown gold lines. Three needles of the hard contact probe station are precisely arranged to form three lines with distinct lengths: L1 (180 μm), L2 (60 μm), and L3 (120 μm) for conductance measurements. (b) Measured I–V diagrams for the three lines indicated in (a) with their corresponding conductance values: L1 (8 mS), L2 (34 mS), and L3 (14 mS).

Here, the conductivity of the gold lines at the edges of the type 4-a and type 4-b substrates is investigated. This examination serves to analyze the electrical properties important for application in neuromorphic networks. A hard contact probe station device was employed for conductivity measurements. Due to the microscopic sizes of the photodeposited gold structures and the limited visibility under the microscope, only three probes were brought into contact with a photodeposited gold line at a given time. The application of a voltage ramp ranging from −5 to +5 V allowed for the recording of current between two probes, as illustrated in Figure 6. Given the narrow width of approximately 5 μm of the gold lines, which closely matches the size of the measurement needle tips, obtaining accurate and reliable contacting imposed experimental challenges. Additionally, the delicate nature of the grown gold lines and their tendency to detachment from the substrate during measurements posed a recurring challenge, necessitating careful handling to preserve the sample integrity. Given the presence of gaps in the grown line of the type 4-a substrates, achieving a uniform and gap-free line also posed a challenge. However, we successfully identified a line with a length of 180 μm and measured its conductance. To ensure a trustworthy comparison, the conductance of a line with an identical length of 180 μm was measured in the type 4-b substrate. The conductances of the lines are 3 mS for the type 4-a substrate and 8 mS for the type 4-b substrate, as determined from the slope of the I–V curves (see Figure S4). Reference conductance measurements on the surface of the TiO_2_ lines without gold confirmed their nonconductive nature.

Additionally, conductance measurements were carried out at varying line lengths for the type 4-b substrate. Three needles of the hard contact probe station were arranged along a line as shown in part a, resulting in three distinct lines indicated as L1, L2, and L3. The I–V diagrams for each of the three lines are demonstrated in Figure 6b. Line L1, situated between the two outer needles with a length of 180 μm, demonstrated a conductance of 8 mS. For line L2, with a length of 60 μm, a conductance value of 34 mS was measured. Meanwhile, line L3, with a length of 120 μm, exhibited a conductance of 14 mS. The approximate values for the lengths are attributed to the substantial size difference between the needle tips and the line width. Analysis of the I–V diagram for these three lines reveals a noteworthy pattern: as the length increases, the conductance decreases, aligning with expectations.

In combination, the SEM, AFM, and EDX images reveal distinct gold growth patterns on the type 4 substrates compared to the other substrate types. While gold growth is observed on the TiO_2_–ITO-coated surface for the other substrate types, type 4 substrates show gold growth only along the edge between the coated and uncoated surface areas. Given that there is no edge growth for the reference substrates with a single ITO layer (type 1), a single 70 nm TiO_2_ layer (type 2), and a single 845 nm TiO_2_ layer, we conclude that the combination of the ITO and TiO_2_ layers is essential for gold growth along the edges. On the other hand, the type 5 substrate, comprising a 6 nm ITO layer and a thick TiO_2_ layer, does not exhibit significantly enhanced edge growth or suppressed surface growth. Here, two effects need to be considered: the electron diffusion distance to the ITO interface and the layer morphology. The illumination is conducted from the top. Considering the TiO_2_ anatase refractive index as
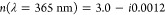
1The absorption coefficient is approximately 400 cm^–1^ at the peak wavelength of 365 nm.^24^ Hence, even with the 845 nm thick TiO_2_ layer, electron generation is expected to occur throughout the depth of the layer. Transfer-matrix simulations for normal-incidence illumination predict a UV-light intensity drop of approximately 10% within the 845 nm TiO_2_ layer. The LED has a spectral half-width of about 9 nm and the absorption increases toward 350 nm. Thus, a larger fraction of the short-wavelength photons is absorbed (50% intensity drop within the 845 nm TiO_2_ layer calculated for an excitation wavelength of 355 nm). Once gold growth starts, part of the light is reflected on the gold and the absorption is decreased in this area. Also, additional scattering effects are expected due to surface roughness and gold nanoparticles that are not included in the simulation.

The distribution of electrons within the TiO_2_ layer is additionally influenced by thin-film interference effects. The wavelength of the UV excitation light in TiO_2_ is approximately 122 nm (365 nm divided by the refractive index). Thin-film interference maxima are expected to have a spatial distance of 61 nm at normal incidence. For the 845 nm TiO_2_ layer, approximately 14 maxima (and minima) in electron generation are expected to pass from top to bottom. The 70 nm TiO_2_ layer just exceeds one period in the spatial interference pattern.

The photogenerated electrons diffuse in the TiO_2_ layer. For the 845 nm thick TiO_2_ layer, the carrier generation occurs at a greater average distance to the ITO interface. Only for electrons reaching the ITO layer, changes are expected in the gold growth. We estimate the diffusion length as the characteristic length as
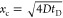
2where the exponent of the one-dimensional diffusion result^25^ is equal to −1. Due to the thin-film geometry and homogeneous illumination, one-dimensional diffusion is a good model. Once the islands of gold start growing, additional effects occur. For analyzing the onset of gold growth, the one-dimensional model is sufficient. Considering, e.g., an electron diffusion constant^26^*D* = 1 × 10^–6^ m^2^/s and a diffusion time of *t*_D_ = 10 ns before recombination, *x*_c_ = 200 nm is obtained.

The expected diffusion length of approximately 200 nm suggests that electrons generated in the 70 nm TiO_2_ layer efficiently pass the Schottky barrier into the ITO. This estimation explains the quenched gold growth on the TiO_2_-coated surface of type 4 substrates. Photogenerated electrons are efficiently collected into the ITO layer and are thus not available for photocatalytic deposition of gold at the surface. Only part of the electrons generated in the 845 nm thick TiO_2_ layer are expected to reach the ITO layer. This is in line with the observed gold growth on the surface of the type 5 substrates.

In addition to suppressing gold growth on the TiO_2_ surface, the ITO sublayer also promotes electron transport to the edge and gold growth along the edge of the microstructured region. Gold growth along the edge starts from gold islands at the edge. The islands show coalescence as they grow, forming a conductive gold line along the edge. Just considering the discussion so far, more electrons are expected to reach the ITO layer for the type 5 substrates than for the type 4 substrates due to the thicker TiO_2_ layer. Thus, for the type 5 substrates, surface and edge growth are expected. Experimentally, only one of the four type 5 substrates exhibits significant edge growth. Therefore, another factor influences gold growth.

We attribute the additional effects to the different deposition methods for the thick 845 nm TiO_2_ layer. Cracking of the layer is desired and induced in the fabrication to allow for the efficient transfer of carriers to the TiO_2_-solution interface. This different layer morphology reduces the diffusion time to the solution and fosters gold growth at the surface. As gold growth at the surface is dominant in the type 5 samples, the carrier transfer to the solution appears additionally enhanced, while the edge growth is suppressed. It needs to be noted that the different TiO_2_ film morphologies will also have an effect on the optical properties and thus on the electron generation rate as a function of depth in the layer. For an enhanced edge growth, a TiO_2_ layer deposition method generating homogeneous layers without cracks appears preferable. This reduces the electron recombination rate and enhances the transfer of electrons to ITO.

By using the proposed approach with an ITO sublayer, all photocatalytically deposited gold contributes to the formation of a conductive line. Compared to simply depositing gold on a line-shaped microstructured TiO_2_ layer, this approach has the advantage that electrons are collected from a larger region, achieving faster coalescence of the conductive line. This effect is clearly observed by comparing the type 4-a and type 5 substrates. For both substrates, the same UV illumination time is used and the type 4-a substrates already have significantly longer conductive segments.

## Conclusions

In summary, our study introduces an approach to grow gold lines along the edges of patterned TiO_2_–ITO substrates through the photocatalytic reduction of precursor ions from HAuCl_4_ solution by UV-stimulated TiO_2_. We further investigated the impact of two different TiO_2_ layer thicknesses—70 and 845 nm—on the gold growth properties. EDX analysis confirmed the composition of the grown line as gold. Comprehensive SEM and AFM analysis of the physical morphology shows a gold line approximately 7 μm wide and 4 μm high. The conductance is approximately 8 mS for a 180 μm long section.

Our main application focus is the on-demand formation of long-range connections in neuromorphic networks. Photodeposition of conductive gold lines along the edges of a photocatalytically active ITO/TiO_2_ thin film stack complements the toolbox of neuromorphic engineering by providing a technical implementation of stimulus-controlled dynamic formation of directional conductive interlinks. With this type of functionality, the grown gold lines show a certain resemblance to the role that axons take in signal transmission in neural networks. For neuromorphic computing, time scales on the order of minutes to hours are suitable to mimic the slow evolution of the topology of biological neuronal networks.

The proposed approach to photocatalytic deposition—photogeneration of electrons in a thin-film layer, collection in a dedicated conductive sublayer, and growth by reduction at a different position—opens opportunities for localized material deposition. It is also promising for other shapes of microstructures, and it will be interesting to investigate how far this effect of localized deposition extends to the nanoscale. While our main aim was to achieve uniform conductive gold lines, the different types of TiO_2_–ITO substrates investigated here are also of high interest for photocatalytic applications as well as for biosensing applications. Charge carriers are collected for localized enhancement of photocatalysis at the ITO/TiO_2_ edges of type 4 substrates. On the other hand, a uniform photocatalytic performance is achieved across the surface of the type 5 substrates. This approach may be utilized for other types of photocatalytic reactions in microreactors. Also, the localized gold microstructures may be functionalized for biosensing allowing for localized sensing.

## Supporting Information Available

Details of the substrates preparation, SEM images of the different types of substrates and their corresponding gold surface coverage evaluation, I–V diagrams of gold lines grown on the two type 4 substrates, and band diagram of the Schottky barrier formed at the TiO_2_–ITO interface (PDF)
