# Supplementary material for: Photocatalytic Edge Growth of Conductive Gold Lines On Microstructured TiO2–ITO Substrates
Source: Langmuir. 2024 Aug 28;40(36):19051–9. doi: 10.1021/acs.langmuir.4c02106 (PMC11394007; doi:10.1021/acs.langmuir.4c02106)
Supplement: Supplementary file 1 — la4c02106_si_001.pdf [file la4c02106_si_001.pdf]

Supporting Information

Photocatalytic edge growth of conductive gold lines on microstructured TiO_2-ITO substrates

Fatemeh Abshari[a], Salih Veziroglu[b,c]. Blessing Adejube[b], Alexander Vahl[b,c,d], Martina Gerken[a,c]*

[a]Chair for Integrated Systems and Photonics, Department of Electrical and Information Engineering, Faculty of Engineering, Kiel University, Kaiserstr. 2, D-24143 Kiel, Germany

[b]Chair for Multicomponent Materials, Department of Materials Science, Faculty of Engineering, Kiel University, Kaiserstr. 2, D-24143 Kiel, Germany

[c]Kiel Nano, Surface and Interface Science KiNSIS, Kiel University, Christian-Albrechts-Platz 4, D-24118 Kiel, Germany

[d]Leibniz Institute for Plasma Science and Technology, Felix-Hausdorff-Str. 2, 17489 Greifswald, Germany

*Email: fa@tf.uni-kiel.de

Table of Contents

Substrate preparation S3
Figure S1: SEM images of the different types of substrates S5
Figure S2: Gold surface coverage evaluation of the different types of substrates........ S7
Figure S3. I-V diagram of a 180-µm long line on the type 4-a and type 4-b substrates........ S8
Figure S4. Schottky barrier formation at the TiO_2-ITO interface S8
References S9

Substrate preparation

As the first step to prepare the substrates, single-side polished 4-inch silicon wafers (111 orientation, Microchemicals GmbH) with a SiO_2 thermal oxide layer (1000 nm thick, wet thermal oxide) were diced into 10×10 mm pieces (Aurotech company, Wafer Dicing DAD3350 model). The resulting substrates were cleaned with acetone and isopropanol (both Sigma-Aldrich) in an ultrasonic bath (Martin Walter Ultraschalltechnik) at grade 9. They were then thoroughly dried using pure nitrogen gas.

A 5-inch lithography mask including reflective chromium structures (Rose Fotomasken) with feature sizes down to 50 μm and different line shapes was used to create microstructures on the substrate via UV photolithography. To enhance adhesion, hexamethyldisilane (HDMS) was first applied to each silicon substrate at 100°C. Next, 200 μL of AZ5214E photoresist (Microchemicals GmbH) was spin coated at 3000 rpm for 30 seconds (ST22model, Robotechnik). The substrates were prebaked on a hot plate at 110°C for 50 seconds. Utilizing a mask aligner (SUSS MicroTec), they were exposed to UV light with an energy of 32 mJ/cm^2 through the designed lithography mask. A reversal bake was performed on a hot plate at 120°C for 2 minutes. Subsequently, a flood UV exposure with an energy of 320 mJ/cm^2 was conducted. The final step in the lithography process was to develop the non-exposed areas during the first illumination, using AZ726 developer (Microchemicals GmbH). Hence, the substrates were immersed in the developer for 50 seconds. To eliminate any remaining developer on the substrates, they were washed with deionized water and subsequently dried with a pure nitrogen gas gun. The schematic of the UV photolithography process is illustrated in Figure 1a.

To deposit the ITO coatings, a 3-inch In_2O_3/SnO_2 (90/10 wt%) target with a purity of 99.99% was used (Kurt J. Lesker). The deposition process was conducted by a sputtering machine with a

tooling factor of 191. Meanwhile, the TiO_2 coatings were deposited using a 3-inch TiO_2 target with a purity of 99.99% (Kurt J. Lesker) by a sputtering machine with a tooling factor of 28. The base pressure in the vacuum chamber was set at 3.6×10^{-7} Pa (3.6×10^{-9} mbar). This low base pressure was crucial for achieving high-quality film deposition and minimizing contamination. During the sputtering of TiO_2 layer, the pressure of the sputtering chamber was maintained at 2.1×10^{-3} mbar. The distance between the titanium target and the substrate during sputtering was approximately 10 cm. This distance was carefully controlled to ensure uniform film deposition across the substrate surface. For lift-off, the substrates were placed vertically in a beaker containing acetone, secured with a holder, and were subjected to ultrasonic agitation for 10 minutes. Subsequently, they were immersed in isopropanol for an additional 5 minutes. The substrates were thoroughly rinsed with deionized water and dried using nitrogen gas. To convert the TiO_2 thin films to anatase phase, the substrates underwent a heat treatment process. The four substrates were heated at 400°C in a muffle furnace for 90 minutes. Following this step, they were promptly cooled using a metal plate.

To achieve thicker TiO_2 with a different morphology, a second TiO_2 sputtering method was exploited for the type 3 and type 5 substrates. To build the type 5 substrate, a 6 nm ITO sublayer was coated by PVD prior to the deposition of an 845 nm ± 20 nm TiO_2 layer using a sputtering system equipped with a DC planar magnetron source (Advanced Energy, MDX 500). Metallic titanium target (Ti-Goodfellow, 99.99%, 5 cm diameter) was used for sputtering, with argon (Ar) and oxygen (O_2) as process and reactive gases, respectively. The substrates were placed into the vacuum chamber and the base pressure was set to about 5×10^{-5} Pa using a turbo molecular pump (Pfeiffer Vacuum, HiPace 400) and a rotary pump (Agilent Technologies, SH-110). An Ar/O_2 gas mixture with a constant ratio of 250:10 sccm was supplied using a precise mass-flow-control system (MKS, Multi Gas Controller 647C) at a DC power of 120 W (BeamTech DC Pulse Power

Supply MPS1500P). The substrates were rotated at 30 rpm to ensure homogeneous film deposition. Following the 2-hour sputtering process, the prepared TiO_2 thin films underwent heat treatment in a muffle furnace at a temperature of 400°C for 90 minutes and then directly quenched in air.

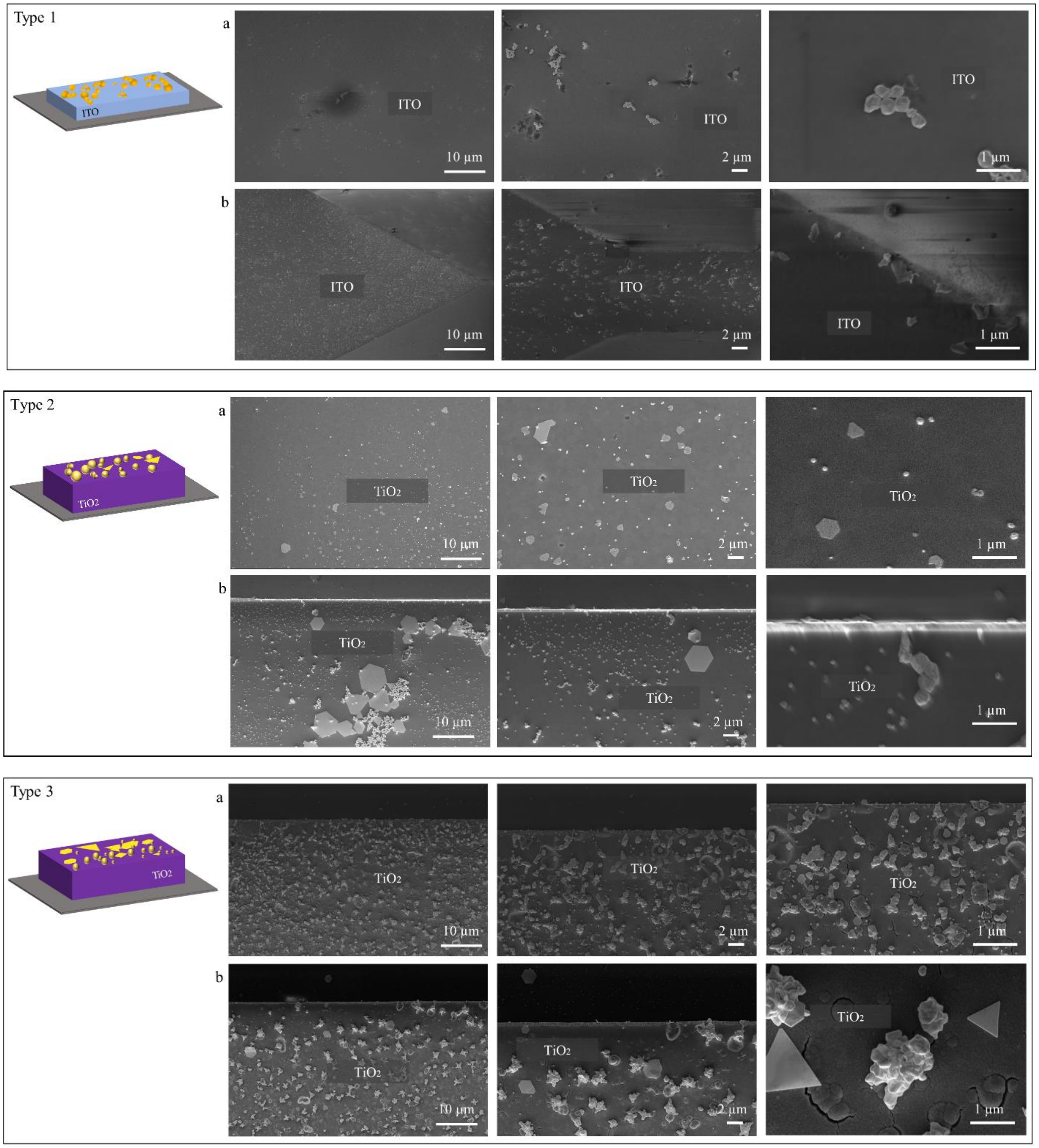

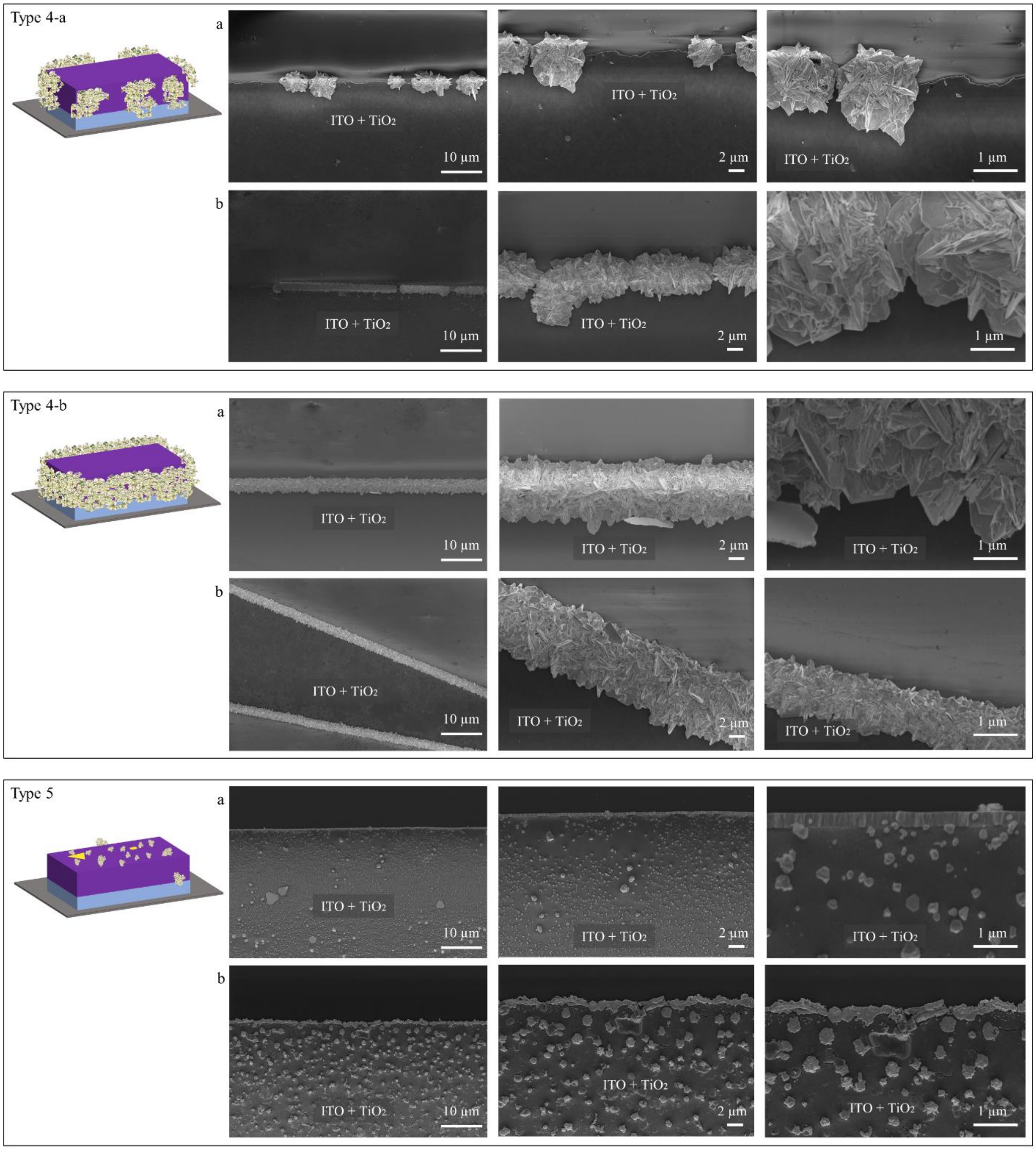

Figure S1. SEM images of the different types of substrates (type 1 – type 5). For each substrate type, two different samples (a and b) are demonstrated for comparison.

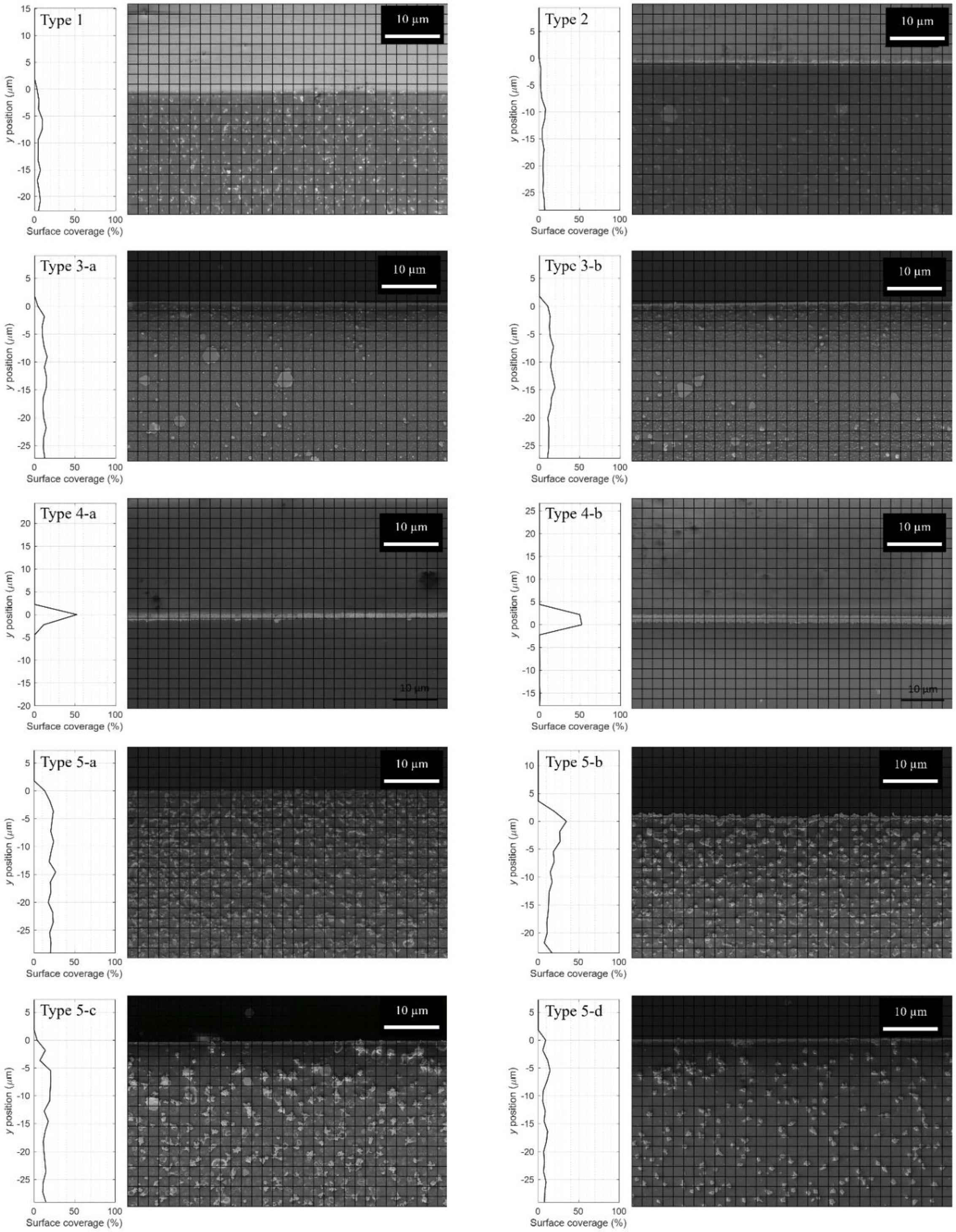

Figure S2. SEM images of the different types of substrates (type 1 – type 5) along with their corresponding gold surface coverage evaluation. Two samples of type 3 and four samples of type 5 substrates are studied and the results are shown for comparison.

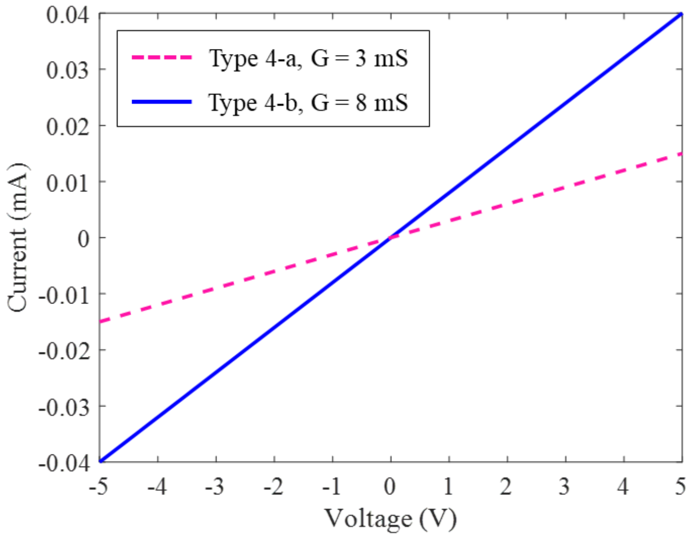

Figure S3. I-V diagram measured for a 180-µm long line on the type 4-a and type 4-b substrates with their corresponding conductance values: type 4-a (3 mS) and type 4-b (8 mS).

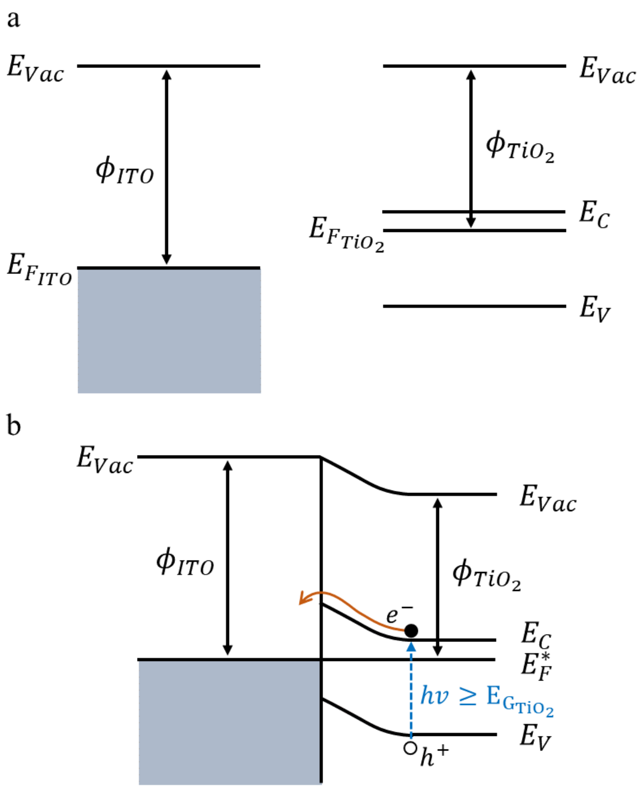

Figure S4. (a) Band diagrams of conductive ITO (left) and semiconductor TiO_2 (right). (b) Since ITO has a higher work function than TiO_2, a Schottky barrier is formed at the TiO_2-ITO interface

[S1]. When the sample is exposed to UV light ($\boldsymbol{hv} \geq \mathbf{E}_{\mathbf{G_{TiO_2}}}$), electron-hole pairs are generated within the TiO_2 layer causing a shift in its Fermi level. To re-establish equilibrium, the photogenerated electrons flow across the junction from TiO_2 to ITO. This results in a continuous flow of photogenerated electrons from TiO_2 towards ITO.

References

[S1] Dai, W.; Wang, X.; Liu, P.; Xu, Y.; Li, G.; Fu, X. Effects of Electron Transfer between TiO_2 Films and Conducting Substrates on the Photocatalytic Oxidation of Organic Pollutants. *J. Phys. Chem. B* **2006**, 110(27), 13470-13476.
